# Age-related macular degeneration – clinical review and genetics update

**DOI:** 10.1111/cge.12206

**Published:** 2013-07-09

**Authors:** R Ratnapriya, E Y Chew

**Affiliations:** a Neurobiology-Neurodegeneration and Repair LaboratoryBethesda, MD, USA; b Division of Epidemiology and Clinical Applications, National Eye Institute, National Institutes of HealthBethesda, MD, USA

**Keywords:** age-related macular degeneration, disease management, exome-chip, GWAS, rare-variant association, risk prediction, whole-exome sequencing, whole-genome sequencing

## Abstract

Age-related macular degeneration (AMD) is the leading cause of central vision impairment in persons over the age of 50 years in developed countries. Both genetic and non-genetic (environmental) factors play major roles in AMD etiology, and multiple gene variants and lifestyle factors such as smoking have been associated with the disease. While dissecting the basic etiology of the disease remains a major challenge, current genetic knowledge has provided opportunities for improved risk assessment, molecular diagnosis and clinical testing of genetic variants in AMD treatment and management. This review addresses the potential of translating the wealth of genetic findings for improved risk prediction and therapeutic intervention in AMD patients. Finally, we discuss the recent advancement in genetics and genomics and the future prospective of personalized medicine in AMD patients.

Age-related macular degeneration (AMD; MIM, 603075) is a late-onset, multifactorial neurodegenerative disease characterized by progressive degeneration of photoreceptors/retinal pigment epithelial complex primarily in macular region of the retina, resulting in irreversible central vision loss. AMD is the major cause of vision loss in individuals 50 years or older in developed countries, affecting nearly 10% of those >65 years of age and affects >25% of those >75 years of age 1. In the United States alone, more than 8 million have intermediate AMD and nearly 2 million have advanced AMD. These numbers are projected to increase by 50% by 2020 2.

Human retina undergoes changes as part of the natural course of aging, resulting in the appearance of ophthalmoscopically visible focal yellow deposition of acellular, polymorphous debris called drusen between the retinal pigment epithelium and Bruch's membrane. Drusen are classified as small (<63 µm in diameter with discrete margins), medium (63–124 µm) or large (>125 µm with indistinct edges, Fig. 1a). Drusen are the characteristic physical signs of AMD, but individuals with drusen alone, particularly small drusen are less likely to develop advanced AMD, especially in the absence of other ocular abnormalities. Focal detachment of the retinal pigment epithelium (RPE), new blood vessel growth between Bruch's membrane and the retina, and outer retinal atrophy are the lesions that can cause loss of central vision in advanced AMD, which can either involve choroidal neovascularization (CNV, Fig. 1b–c) or be non-neovascular or geographic atrophy (GA, Fig. 1d). AMD patients display a broad spectrum of clinical characteristic based upon drusen size and AMD pigmentary abnormalities, both hypopigmentation and hyperpigmentation. Age-Related Eye Disease Study (AREDS) has developed a simplified five-step severity scale to define risk categories for development of advanced AMD 3.

**Fig. 1:**
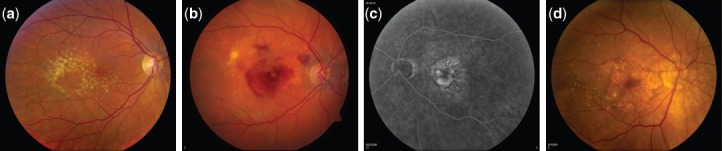
Fundus photographs at different stages of age-related macular degeneration (AMD) progression. (**a**) Large and intermediate drusen at intermediate stage of AMD. (**b**) Neovascular AMD—right eye with evidence of sub-retinal fluid, hemorrhage, and hard exudate in the presence of choroidal neovascularization. (**c**) Fluorescein angiography of the neovascular AMD—Left eye showing the hyperfluorescence of the fluorescein angiogram corresponding to the area of the choroidal neovascularization. (**d**) Central geographic atrophy—Right eye with evidence of geographic atrophy involving the center of the fovea with evidence of large drusen temporally.

## Risk factors in AMD

AMD is a multifactorial disease, typically caused by many genetic variants, each with modest effect on the risk and also influenced by non-genetic/environmental factors, such as diet and smoking 4. Long-term epidemiological studies have identified valuable information on the prevalence, incidences, natural history and associated risk factors of AMD 5, 6. Large, soft drusen associated with AMD pigmentary abnormalities have been associated with an increased risk of progression to advanced AMD. Neovascular AMD is the more common cause of blindness although it may account for smaller percentage of AMD than GA. Aging and smoking have been demonstrated to be the most consistent non-genetic risk factors. Increasing pack years of cigarettes smoked is directly associated with increasing risk of AMD, usually with a doubling of the risk when comparing smokers to those who have never smoked 7. In addition, cardiovascular risk factors, such as hypertension and hyperlipidemia have been inconsistently associated with AMD risk as well. Elevated serum lipids (triglycerides) were associated with increasing intermediate AMD in one series while another population found no association with serum lipids 8, 9. A 10-year longitudinal study, Multi-ethnic Study of Atherosclerosis (MESA) examined four racial/ethnic groups and reported lower prevalence of AMD in blacks than in whites with overall prevalence varying from 2.4% in African Americans, 4.2% in Hispanics, 4.6% in Chinese compared with 5.4% in whites 6.

Genetic contribution to the development of AMD has been established over the years through familial aggregation studies, twin studies, and segregation analyses (Klaver, #75; Meyers #76 10). Early linkage studies on smaller families identified multiple genetic loci at chromosomes 1q25-31, 9p13, 9p24, 10q26, 15q21 and 17q25 11–14 (see additional references, 1–3 in supplementary file). Genome-wide association studies (GWAS) and candidate association studies have further contributed significantly in identifying the risk loci for AMD and have implicated genes—*CFH* 15–19, *C3* 20, 21, *C2-CFB* 22, *CFI* 23, a region on chromosome 10 with *HTRA1*/*LOC387715*/*ARMS2* 24–26, *CETP* 27, *TIMP3* 27, *LIPC* 28, *VEGFA* 29, *COL10A1* 29, *TNFRSF10A* 30, and *APOE* 31–33 with AMD (Table 1).

**Table 1:** Summary of age-related macular degeneration AMD associated genes/loci as of April 2013^a^

DNA marker	Nearby gene(s)	Genetic method	Pathway	Validation status
rs1800553, rs1800555	*ABCA4*	Candidate gene	Clearance of all-trans-retinal aldehyde from photoreceptors	Confirmed
rs6795735	*ADAMTS9*	Meta-analysis	Unknown	Tentative
rs429358, rs7412	*APOE*	Candidate gene/GWAS	Transport and metabolism of lipoproteins	Confirmed
rs10490924 (*ARMS2*), rs11200638 (*HTRA1*)	*ARMS2*/*HTRA1*	Linkage/GWAS	Unknown	Confirmed
rs9542236	*B3GALTL*	Meta-analysis	Unknown	Tentative
rs9332739 (*C2*), rs4151667 (*CFB*)	*C2*/*CFB*	Candidate gene/GWAS	Complement pathway	Confirmed
rs2230199	*C3*	Candidate gene/GWAS	Complement pathway	Confirmed
rs3764261	*CETP*	GWAS	Transport and metabolism of lipoproteins	Confirmed
rs1061170, rs10737680	*CFH*	Linkage/GWAS/candidate gene	Complement pathway	Confirmed
84 K bp deletion	*CFHR1*/*CFHR3*	Candidate gene	Possibly complement pathway	Tentative
rs2285714	*CFI*	Candidate gene/GWAS	Complement pathway	Confirmed
rs13095226, rs13081855	*COL8A1*/*FILIP1L*	GWAS	Extracellular/collagen matrix pathway	Confirmed
Missense variants	*FBLN5*	Candidate gene	Extracellular matrix pathway	Confirmed
rs1999930, rs3812111	*FRK*/*COL10A1*	GWAS	Extracellular/collagen matrix pathway	Confirmed
rs3130783	*IER3-DDR1*	Meta-analysis	Unknown	Tentative
rs493258, rs10468017, rs920915	*LIPC*	GWAS	Transport and metabolism of lipoproteins	Confirmed
rs8017304	*RAD51B*	Meta-analysis	Unknown	Tentative
rs1713985	*REST-C4orf14-POLR2B-IGFBP7*	GWAS	Unknown	Tentative
rs8135665	*SLC16A8*	Meta-analysis	Unknown	Tentative
rs334353	*TGFBR1*	Meta-analysis	Angiogenesis	Tentative
rs9621532, rs5749482	*TIMP3*	GWAS	Degradation of the extracellular matrix	Confirmed
rs13278062	*TNFRSF10A*	GWAS	Unknown	Confirmed
rs2071277	*TNXB-FKBPL-NOTCH4*	GWAS	Notch-signaling pathways	Tentative
rs4711751, rs943080	*VEGFA*	Candidate gene/GWAS	Angiogenesis	Confirmed

GWAS, genome-wide association analysis.

a Confirmed, when identified in two or more studies; Tentative, when reported in only one study and pending independent replication(s)

## Current status of AMD genetics

AMD remains one of the well-characterized complex traits with multiple loci identified, accounting for substantial heritability. In an effort to identify additional loci with smaller effects on risk, which could have been missed in the GWAS owing to the sample size and adjustment for multiple testing, combined analysis of the data from multiple GWAS studies (meta-analysis) is emerging as an important next step to explain unaccounted heritability. A recent international collaborative efforts on meta-analysis of AMD-GWAS from 18 centers, involving >17,000 AMD cases and 60,000 matched controls of European and Asian ancestry, has revealed 19 AMD loci, including 7 novel ones near the genes *COL8A1/FILIP1L*, *IER3/DDR1*, *SLC16A8*, *TGFBR1*, *RAD51B*, *ADAMTS9*, and *B3GALTL* 34.

Identification of multiple genetic loci have implicated several important biological pathways, such as: complement pathway, cholesterol and lipid metabolism pathway, extracellular/collagen matrix pathway, oxidative stress pathway, and angiogenesis signaling pathway in AMD pathogenesis 35–37, providing opportunity to understand the disease mechanism (Table 1). However, associated variants may not be causal and further genetic dissection of susceptibility loci is warranted to identify the underlying disease-causing variants. More importantly, functional characterization of genes at associated loci is highly desirable to gain insights into biological relevance with AMD for complete understanding of AMD disease pathogenesis.

## AMD disease management and therapy

With limited treatment option available, preventing the development and retarding the disease progression to minimize the vision loss remain high priorities in AMD management. The AREDS established that antioxidant vitamin and mineral supplementation (AREDS formulation) consisting of β-carotene (15 mg), vitamins C (500 mg) and E (400 IU), and zinc (as zinc oxide 80 mg), along with copper (as cupric oxide 2 mg) slowed progression of AMD in individuals at high risk of developing advanced AMD 38. Recent analyses demonstrate that the beneficial effects are restricted mostly to preventing the progression to neovascular AMD 39. Based upon the observational data from AREDS 40, 41 and other studies (see additional references, 4–10 in supplementary file), the AREDS 2, a randomized controlled clinical trial, was designed to evaluate whether adding lutein (10 mg)/zeaxanthin (2 mg) and/or omega-3 long-chain polyunsaturated fatty acids, docosahexaenoic acid (DHA-350 mg) and its precursor, eicosapentaenoic acid (EPA-650 mg) will affect the rates of progression to advanced AMD 42. An additional randomization included the evaluation of eliminating beta-carotene and lowering the dose of zinc in the original AREDS formulation. The results of the AREDS2 showed that adding omega-3 fatty acids to the AREDS supplements were neither beneficial nor harmful 43. Adding lutein/zeaxanthin to the AREDS formulation resulted in additional beneficial effect of about 20% beyond the effects of AREDS formulation in reducing the risk of progressing to advanced AMD. Furthermore, beta-carotene was associated with an increased risk of lung cancer, mostly in former smokers. In both AREDS and AREDS2, 50% of persons with AMD also were former smokers. The recommendations from the AREDS2 results include the elimination of beta-carotene, with the substitution of lutein/zeaxanthin in the AREDS formulation.

Anti-vascular endothelial growth factor (VEGF) therapy is the most effective and widely used treatment for neovascular AMD. VEGF is a secreted endothelial-specific mitogen, which acts as a key regulator of angiogenesis and vascular permeability. Numerous studies have implicated inflammation as an important player in pathogenesis of AMD 44 and high expression of VEGF has been recognized as an important factor promoting neovascularization in wet AMD 45 (see additional references, 11–13 in supplementary file). Current approaches to inhibit VEGF involved the development of humanized monoclonal antibodies, bevacizumab (Avastin®; Genentech, Inc, South San Francisco, CA) and its derivative, ranibizumab (Lucentis®; Genentech, Inc), which neutralizes all active forms of VEGF and thus incapacitating the effect of VEGF on increased vascular permeability and presumably, angiogenesis. Newer anti-VEGF therapy includes aflibercept (VEGF Trap-eye; Regeneron Pharmaceuticals, Tarrytown, NY; Bayer Plc), which is a recombinant human fusion protein acting as a soluble decoy receptor for VEGF family. Usage of these intravitreal drugs has revolutionized treatment of neovascular AMD, but not all participants respond to these intravitreal anti-VEGF therapies with 40% gaining vision. Further research is still required both to increase the efficacy for all patients and to improve on the drug delivery system to reduce the burden of frequent intravitreal injections. Currently, these treatments are associated with enormous monetary and human cost. Vision loss has also been observed following anti-VEGF therapy, which may be secondary to GA. No effective treatment is available for GA.

## Implication of genetic finding on AMD risk prediction and progression

Accurate prediction of genetic predisposition and progression hold promise for potential preventive measurements in AMD patients. Post-GWAS era has led to a number of studies exploring the effect(s) of genetic variants in prediction genetics risk or progression. Because the disease symptoms for AMD does not appear until sixth decade of patient's life, early detection of individuals at risk may allow early interventions and additional motivation in patients for adhering to lifestyle changes which include smoking cessation and increasing dietary intake of fish and vegetables that contain lutein and zeaxanthin. In fact, in the Rotterdam Study, diets high in antioxidant properties were found to reduce the risk of development of early AMD in persons who had high genetic risk for developing AMD 46.

Further research is required to find effective treatment/preventive regimes. Multiple studies have assessed the role of genetic variants on AMD risk and progression, especially with *CFH* and *ARMS2* genes, the two major susceptibility genes for AMD. *CFH* Y402H heterozygotes allele conferred 4.6-fold increased risk for AMD and the homozygotes a 7.4-fold increased risk, as compared with the homozygous non-risk genotype 17. Individuals heterozygous for *ARMS2* A69S allele confer ∼2.7-fold increased risk of AMD compared with wild-type homozygous allele, whereas a 8.2-fold increased risk is associated with the homozygous risk allele 25. An additive effect of *CFH* Y402H and *LOC387715* A69S is seen with 50-fold increase in the risk of AMD in subjects homozygous for both risk alleles 47.

AMD is likely to be manifestation of several different conditions. Phenotyping of AMD continues to be enhanced with improved technology both in imaging and measures of retinal function. Genotyping will also lead to better phenotyping in the future. A summary of some of the current genetic associations with different lesions and subtypes has been summarized in the supplementary material.

*CFH* and *ARMS2* represent rather a unique example in complex traits as these two susceptibility loci alone contribute substantially to the AMD heritability. This has resulted in occasional success with risk prediction and progression in few studies. However, these approaches lack the sensitivity and specificity with most of the individuals having middle range of AMD risk and thus have little diagnostic value 35, 48. Moreover, there are multiple additional genes with small effect size on AMD susceptibility. Thus testing genetic risk using single susceptibility gene variants will have limited predictive value. Calculating the risk based on combination of all risk allele seems more appropriate but requires robust estimates of risk scores for each of the loci. Additionally, understanding the interplay with non-genetic environmental factors, such as smoking and diet, as well as clinical features of the retina, is essential for precise risk prediction. Some recent studies have made efforts in combining the multiple genetic variants alone 49 or with non-genetic rick factors in predictive modeling 10 (see additional references,14-15 in supplementary file). These models need further validation in long-term prospective studies and in young at-risk population. A comprehensive understanding of genetic architecture of AMD is desirable for realizing the true potential of genetic risk prediction.

## Pharmacogenomics and biomarker discovery

Testing the effect of genetic variants in patients on the response to drug therapy, both in terms of efficacy and adverse drug reactions is referred to as pharmacogenomics. Anti-VEGF therapy is effective, but need repeated intraocular injection and poses threat for long-term adverse events because of systemic penetration 50. The comparison of AMD treatment (CATT) trials showed no clinical important differences between ranibizumab and bevacizumab 51, 52. However, it has been suggested that there is individual variation in the response to treatment. These variations have often been attributed to genetic variation, leading to few small-scale pharmacogenetic testing studies in AMD patients using single or few gene markers (*CFH*, *ARMS2*, and *VEGFA*) 53–55. *CFH* and *ARMS2* variants have also been investigated to predict treatment response to AREDS-type nutritional supplementation with antioxidants and zinc in AREDS participants 56. Investigators have reported a potential pharmacogenetic interaction (p = 0.03) between *CFH* Y402H genotype and supplementation with antioxidants and zinc. While limited success has been achieved in few pharmacogenetic studies with *CFH* and *ARMS2* variants, prospective randomized, control trials should be conducted to substantiate these associations. Moreover, drug metabolism and variation in drug response may result from interaction of several genes and multiple polymorphisms. Thus testing few markers association alone will not yield much meaning into clinical practice.

Genetic and pathophysiological data on AMD have provided compelling evidence of involvement of inflammatory, complement and high-density lipoprotein (HDL) cholesterol pathway in AMD pathogenesis. Several studies have identified association of serum-based protein markers, such as C-reactive protein, complement factors, circulating VEGF, antiretinal antibodies, lipid levels and carboxyethylpyrrole (CEP) antibodies as biomarkers of AMD. However, there is no clear evidence for any serum biomarker as effective predictor of AMD development or progression 57.

## Future of genetic medicine in AMD

Genomic finding in AMD has potential relevance for genetic based therapeutics in AMD patients, but needs more comprehensive understanding of genetic architecture of the disease. GWAS has provided a basic framework with identification of major common susceptibility genetic variants. Further efforts are needed to identify additional genes/pathways with small or large effect on AMD risk. Identification of susceptibility variants among different AMD sub-phenotypes is also important, but has not been fully evaluated either because of small sample size or lack of detailed phenotyping and clinical data. Future large-scale meta-analysis needs to take these factors into account for identification of subtype specific genetic loci.

While GWASs have played critical role in identifying common risk variants, it has failed to explain the complete genetic heritability in complex diseases 58. Missing heritability has been attributed to many factors, including additional common variants with small effect on risk, structural or copy-number variations, epigenetic modifications, and rare variants 58. Recent advancement of next-generation sequencing technologies (NGS) has made systematic identification of rare allele possible through whole exome, genome and targeted re-sequencing at a reasonable cost 59, 60. NGS has been successfully applied for disease gene identification in Mendelian traits 61 and slowly researchers are turning their attention now to understand the extent to which rare genetic variation underpins the heritability in complex traits 62, 63 (see additional references,16-20 in supplementary file). Recently sequencing of 106.7-kb region around *CFH* gene has identified a rare penetrant variant, R1210C in AMD patients 64. Another functional allele, G119R has been reported in *CFI* that confers high risk to AMD 65. As these variants are rare, they are likely to be harbored only by a fraction of patients and sometimes will even have restricted geographic distribution. Thus such studies require a large sample size to achieve the statistical power for detecting rare-variant association and carefully matched cases-control cohorts 66. Analysis of extreme phenotypes as well as families with multiple affected members can serve as suitable alternative in the absence of large sample size for identifying AMD associated rare-variants and candidates. In many instances, genes identified in heritable forms of macular dystrophies, such as *TIMP3* 67, *ABCA4* 68 have been associated with AMD 27, 69. Thus performing whole-exome or genome sequencing in families with macular dystrophies and in rare instances AMD families can help identify novel candidates genes for macular degeneration.

Genotyping rare variants identified in previous sequencing studies using custom genotyping chips offer an economical and rapid alternative for testing common disease-rare variant hypothesis 58, 62. Massive cataloguing of rare variants through 1000 genome 70 and NHLBI exome sequencing projects have been conducted in the last few years 71. This has propelled the design of a second generation of genotyping array for testing the association of rare variants in complex traits, popularly known as exome-chip 66. This approach has the limitation of not identifying novel variants, but nevertheless provides economical platform for testing association with rare variants.

Advances in the NGS technology and rapidly declining cost of sequencing promise an exponential phase for discovery of rare/common variants in AMD that can play prominent role in future genetic medicine. Development of technical infrastructure, such as data representation, storage and incorporation into electronic medical records as well as standardized guideline for NGS protocols for data analysis is a critical aspect of clinical genomics. Ethical issues related to ownership and privacy of genomic data and handling information of incidental or secondary findings needs consideration in personal genomics as well. However, major challenge lies in understanding the functional significance of the variants, their relationship with each other and to epigenetic and environmental contributions. This will be important for planning any future therapeutic interventions or risk predictions in AMD patients. The practical therapeutic approach for complex disease like AMD is likely to combine multiple factors, involving combination of diet, lifestyle and improved pharmacologic interventions based on wealth of genetic information.

## Supporting Information

The following Supporting information is available for this article:

**Appendix S1.** Genetic associations with different retinal lesions and subtypes of advanced AMD.
