# Supplementary material for: Age-related macular degeneration – clinical review and genetics update
Source: Clin Genet. 2013 Jul 9;84(2):160–6. doi: 10.1111/cge.12206 (PMC3732788; doi:10.1111/cge.12206)
Supplement: Supplementary file 1 [file cge0084-0160-SD1.doc]

**Supplementary information**

**Genetic associations with different lesions and subtypes of AMD**

**Basal Laminar Drusen**

Drusen characteristics have been evaluated for both prognostic factors as well as genetic associations. These include basal laminar or “cuticular drusen” drusen, which have been known to be genetic in origin as it occurs as an early onset disease with multiple drusen of uniform size between 25 to 75 microns in diameter. On fluorescein angiography, the basal laminar drusen have a “stars-in-the-sky” pattern. This form of drusen is associated with increased risk of the development “vitelliform” lesions in the macula that lead to severe vision loss later in life. Recent genetic analyses suggest that affected individuals may all carry the Y402H AMD risk variant of *CFH* .

**Reticular Pseudodrusen**

Reticular pseudodrusen has fueled a number of investigations because of the potential increased likelihood of association with progression to advanced AMD. Although this type of drusen was recognized by 1990 by Mimoun, our understanding of this condition is still limited. A uniform definition of the condition has not been established. Although the recent developments in ophthalmic imaging have enhanced the studies of reticular pseudodrusen and genetic association studies, hypotheses as to the origin, drusen composition, and location of the pathology within the retina remain controversial. Evaluations of a population-based study demonstrated that the prevalence of reticular pseudodrusen was increased in persons with homozygous (CC) or heterozygous (TC) for *CFH* Y402H than those without this variant . Others have suggested that *CFH* Y402H risk variant was found to be significantly associated with the lack of reticular drusen. The risk of reticular pseudodrusen was found by another group to be associated with the *ARMS2* A69S risk allele and has been confirmed in a Japanese population. However, a further investigation in this area is warranted.

**Progression of Drusen to Neovascular AMD or Geographic Atrophy:**

There is no consensus regarding the genetic association with progression to the two advanced forms of AMD. Investigators have reported relative contributions of various genetic variants to the development of neovascular AMD or geographic atrophy, though no clear pattern has been identified. For example, homozygosity for *CFH* Y402H polymorphism was found in one series to be associated with neovascular AMD and specifically for those with classic neovascularization, not occult, or a mix of classic and occult, or retinal angiomatosis proliferation. Another group of investigators evaluated the association of *CFH* and *ARMS2* with different forms of neovascular AMD. They also evaluated significance of *C2*/*CFB* variants in AMD and polypoidal vasculopathy . Other researchers have attempted to evaluate the genetic associations with progression and have developed models. Some found that various genetic variants are associated with progression to either neovascular AMD or geographic atrophy while entirely different genes are responsible for the development of drusen. This has to be further evaluated.

1. Boon CJ, Klevering BJ, Hoyng CB et al. Basal laminar drusen caused by compound heterozygous variants in the CFH gene. Am J Hum Genet 2008: 82: 516-523.

2. Klein R, Meuer SM, Knudtson MD et al. The epidemiology of retinal reticular drusen. Am J Ophthalmol 2008: 145: 317-326.

3. Smith RT, Merriam JE, Sohrab MA et al. Complement factor H 402H variant and reticular macular disease. Arch Ophthalmol 2011: 129: 1061-1066.

4. Ueda-Arakawa N, Ooto S, Nakata I et al. Prevalence and genomic association of reticular pseudodrusen in age-related macular degeneration. Am J Ophthalmol 2013: 155: 260-269 e262.

5. Wegscheider BJ, Weger M, Renner W et al. Association of complement factor H Y402H gene polymorphism with different subtypes of exudative age-related macular degeneration. Ophthalmology 2007: 114: 738-742.

6. Hayashi H, Yamashiro K, Gotoh N et al. CFH and ARMS2 variations in age-related macular degeneration, polypoidal choroidal vasculopathy, and retinal angiomatous proliferation. Invest Ophthalmol Vis Sci 2010: 51: 5914-5919.

7. Nakata I, Yamashiro K, Yamada R et al. Significance of C2/CFB variants in age-related macular degeneration and polypoidal choroidal vasculopathy in a Japanese population. Invest Ophthalmol Vis Sci 2012: 53: 794-798.

8. Yu Y, Reynolds R, Rosner B et al. Prospective assessment of genetic effects on progression to different stages of age-related macular degeneration using multistate Markov models. Invest Ophthalmol Vis Sci 2012: 53: 1548-1556.

9. Farwick A, Wellmann J, Stoll M et al. Susceptibility genes and progression in age-related maculopathy: a study of single eyes. Invest Ophthalmol Vis Sci 2010: 51: 731-736.

**Additional references**

1. Majewski J, Schultz DW, Weleber RG et al. Age-related macular degeneration--a genome scan in extended families. Am J Hum Genet 2003: 73: 540-550.
2. Seddon JM, Santangelo SL, Book K et al. A genomewide scan for age-related macular degeneration provides evidence for linkage to several chromosomal regions. Am J Hum Genet 2003: 73: 780-790.
3. Weeks DE, Conley YP, Mah TS et al. A full genome scan for age-related maculopathy. Hum Mol Genet 2000: 9: 1329-1349.
4. Swenor BK, Bressler S, Caulfield L et al. The impact of fish and shellfish consumption on age-related macular degeneration. Ophthalmology 2010: 117: 2395-2401.
5. Seddon JM, Rosner B, Sperduto RD et al. Dietary fat and risk for advanced age-related macular degeneration. Arch Ophthalmol 2001: 119: 1191-1199.
6. Seddon JM, Cote J, Rosner B. Progression of age-related macular degeneration: association with dietary fat, transunsaturated fat, nuts, and fish intake. Arch Ophthalmol 2003: 121: 1728-1737.
7. Seddon JM, Ajani UA, Sperduto RD et al. Dietary carotenoids, vitamins A, C, and E, and advanced age-related macular degeneration. Eye Disease Case-Control Study Group. JAMA 1994: 272: 1413-1420.
8. Augood C, Chakravarthy U, Young I et al. Oily fish consumption, dietary docosahexaenoic acid and eicosapentaenoic acid intakes, and associations with neovascular age-related macular degeneration. Am J Clin Nutr 2008: 88: 398-406.
9. Risk factors for neovascular age-related macular degeneration. The Eye Disease Case-Control Study Group. Arch Ophthalmol 1992: 110: 1701-1708.
10. Tan JS, Wang JJ, Flood V et al. Dietary antioxidants and the long-term incidence of age-related macular degeneration: the Blue Mountains Eye Study. Ophthalmology 2008: 115: 334-341.
11. Barouch FC, Miller JW. The role of inflammation and infection in age-related macular degeneration. Int Ophthalmol Clin 2007: 47: 185-197.
12. Kvanta A, Algvere PV, Berglin L et al. Subfoveal fibrovascular membranes in age-related macular degeneration express vascular endothelial growth factor. Invest Ophthalmol Vis Sci 1996: 37: 1929-1934.
13. Kliffen M, Sharma HS, Mooy CM et al. Increased expression of angiogenic growth factors in age-related maculopathy. Br J Ophthalmol 1997: 81: 154-162.
14. Chen Y, Zeng J, Zhao C et al. Assessing susceptibility to age-related macular degeneration with genetic markers and environmental factors. Archives of ophthalmology 2011: 129: 344-351.
15. Seddon JM, Reynolds R, Maller J et al. Prediction model for prevalence and incidence of advanced age-related macular degeneration based on genetic, demographic, and environmental variables. Investigative ophthalmology & visual science 2009: 50: 2044-2053.
16. Metzker ML. Sequencing technologies - the next generation. Nat Rev Genet 2010: 11: 31-46.
17. Bamshad MJ, Ng SB, Bigham AW et al. Exome sequencing as a tool for Mendelian disease gene discovery. Nat Rev Genet 2011: 12: 745-755.
18. Kiezun A, Garimella K, Do R et al. Exome sequencing and the genetic basis of complex traits. Nature genetics 2012: 44: 623-630.
19. Pritchard JK. Are rare variants responsible for susceptibility to complex diseases? Am J Hum Genet 2001: 69: 124-137.
20. Cirulli ET, Goldstein DB. Uncovering the roles of rare variants in common disease through whole-genome sequencing. Nature reviews Genetics 2010: 11: 415-425.
